# Identification and Characterization of the Amphioxus Lck and Its Associated Tyrosine Phosphorylation-Dependent Inhibitory LRR Receptor

**DOI:** 10.3389/fimmu.2021.656366

**Published:** 2021-06-03

**Authors:** Jiatao Zhou, Zhihui Xiao, Yanli Zhan, Xuemei Qu, Sisi Mou, Chong Deng, Tianxiang Zhang, Xin Lan, Shengfeng Huang, Yingqiu Li

**Affiliations:** Guangdong Province Key Laboratory of Pharmaceutical Functional Genes, State Key Laboratory of Biocontrol, School of Life Sciences, Sun Yat-sen University, Guangzhou, China

**Keywords:** amphioxus, lymphocyte-specific tyrosine kinase (Lck), tyrosine phosphorylation-dependent inhibitory immunoreceptor, leucine rich repeat receptor (LRR), amphioxus Lck associated LRR (BbLcLRR), tyrosine phosphorylation, immunoreceptor tyrosine-based inhibitory motif, TCR-mediated T cell activation

## Abstract

Amphioxus (e.g., *Branchiostoma belcheri*, Bb) has recently emerged as a new model for studying the origin and evolution of vertebrate immunity. Mammalian lymphocyte-specific tyrosine kinase (Lck) plays crucial roles in T cell activation, differentiation and homeostasis, and is reported to phosphorylate both the ITIM and ITSM of PD-1 to induce the recruitment of phosphatases and thus the inhibitory function of PD-1. Here, we identified and cloned the amphioxus homolog of human Lck. By generating and using an antibody against BbLck, we found that BbLck is expressed in the amphioxus gut and gill. Through overexpression of BbLck in Jurkat T cells, we found that upon TCR stimulation, BbLck was subjected to tyrosine phosphorylation and could partially rescue Lck-dependent tyrosine phosphorylation in Lck-knockdown T cells. Mass spectrometric analysis of BbLck immunoprecipitates from immunostimulants-treated amphioxus, revealed a BbLck-associated membrane-bound receptor LRR (BbLcLRR). By overexpressing BbLcLRR in Jurkat T cells, we demonstrated that BbLcLRR was tyrosine phosphorylated upon TCR stimulation, which was inhibited by Lck knockdown and was rescued by overexpression of BbLck. By mutating single tyrosine to phenylalanine (Y-F), we identified three tyrosine residues (Y539, Y655, and Y690) (3Y) of BbLcLRR as the major Lck phosphorylation sites. Reporter gene assays showed that overexpression of BbLcLRR but not the BbLcLRR-3YF mutant inhibited TCR-induced NF-κB activation. In Lck-knockdown T cells, the decline of TCR-induced IL-2 production was reversed by overexpression of BbLck, and this reversion was inhibited by co-expression of BbLcLRR but not the BbLcLRR-3YF mutant. Sequence analysis showed that the three tyrosine-containing sequences were conserved with the tyrosine-based inhibition motifs (ITIMs) or ITIM-like motifs. And TCR stimulation induced the association of BbLcLRR with tyrosine phosphatases SHIP1 and to a lesser extent with SHP1/2. Moreover, overexpression of wild-type BbLcLRR but not its 3YF mutant inhibited TCR-induced tyrosine phosphorylation of multiple signaling proteins probably *via* recruiting SHIP1. Thus, we identified a novel immunoreceptor BbLcLRR, which is phosphorylated by Lck and then exerts a phosphorylation-dependent inhibitory role in TCR-mediated T-cell activation, implying a mechanism for the maintenance of self-tolerance and homeostasis of amphioxus immune system and the evolutionary conservatism of Lck-regulated inhibitory receptor pathway.

## Introduction

The amphioxus, the basal chordate, occupies the evolutionary position between invertebrates and vertebrates and serves as an excellent animal model to unveil the evolutionary enigma of the vertebrate adaptive immune system (1–3). The discovery of the proto-MHC region in the amphioxus genome, identification of monocyte-like cells containing typical large reniform nuclei in the amphioxus (4, 5), and the demonstration that RAG1/2-like protein in the amphioxus derived from the ProtoRAG gene *via* terminal inverted repeat (TIR)-dependent transposon excision can degrade both DNA and RNA (6, 7), suggest that the immune system of the amphioxus is much more complicated than previously thought (4, 6, 7). Inhibitory immunoreceptors play critical roles in mediating self-tolerance to maintain appropriate immune responses. The origin and evolution of inhibitory signaling are not well understood.

Lymphocyte-specific kinase (Lck), a member of the Src superfamily, is majorly expressed in T lymphocytes (8). Lck is stably associated with CD4 and CD8 coreceptors of TCR, which helps initiate signaling (9, 10). After TCR and coreceptors engagement with cognate antigens, Lck is activated and phosphorylates the tyrosine residues on the immunoreceptor tyrosine-based activation motifs (ITAMs) on the CD3ζ and CD3γ, δ, and ε subunits of the TCR complex. The phosphorylated ITAM tyrosine residues of CD3ζ serve as docking sites to recruit and activate the tyrosine kinase Zap70, which then phosphorylates the adaptor protein LAT (linker for activation of T cells) at multiple tyrosine residues. Phosphorylated LAT nucleates multiprotein signaling complexes, leading to T-cell activation (10–13). In addition to phosphorylate ITAMs, Lck can also phosphorylate the ITIMs of HLA-specific killer cell inhibitory receptors (KIR) in NK and T cells (14), and phosphorylate both the ITIM and immune receptor tyrosine–based switch motif (ITSM) of programmed cell death–1 (PD-1) to activate its inhibitory function in T cells (15).

Lck activity is tightly regulated by two tyrosine residues, Y394 and Y505, with activating and inhibitory function, respectively (16, 17). Phosphorylation of Y394 in the kinase domain of Lck stabilizes its activation loop in an active conformation, whereas interaction with SH2 domain of Lck *via* its phosphorylated Y505 residue induces an occluded or closed conformation (18, 19). As a key component in initiating T-cell signaling, phosphorylation of Lck inhibitory Y505 by C-terminal Src kinase (CSK) decreases CD3ζ phosphorylation (20–23). The Lck-deficient T cell line does not induce phosphorylation downstream signaling activation (24, 25).

In this study, we identified amphioxus Lck and MS analysis of BbLck immunoprecipitates from the gut cells, revealing an inhibitory immunoreceptor BbLcLRR. BbLck is structurally and functionally highly conserved with human Lck. BbLcLRR interacts with and is phosphorylated by both BbLck and human Lck. Upon TCR stimulation, overexpressed BbLcLRR is phosphorylated at Y539, Y655, and Y690 by Lck in Jurkat TAg T cells. By simultaneously mutating these three tyrosine residues to phenylalanine (F) residues to construct BbLcLRR-3YF, we analyzed the function of BbLcLRR wild-type and BbLcLRR-3YF mutant in TCR signaling. Overexpression of BbLcLRR inhibited TCR-induced activation of the transcription factor NF-κB, whereas overexpression of BbLcLRR-3YF relieved its inhibitory effect. Moreover, upon TCR stimulation, the decline in IL-2 production with Lck knockdown could be rescued by BbLck overexpression, but was further inhibited by co-overexpression with BbLcLRR but not with BbLcLRR-3YF. TCR stimulation induced the association of BbLcLRR with tyrosine phosphatases SHIP1 and SHP1/2. And BbLcLRR seems to inhibit TCR-induced tyrosine phosphorylation by recruiting SHIP1. In summary, upon TCR stimulation, Lck or BbLck phosphorylates BbLcLRR, which in turn inhibits T-cell activation. Thus, BbLcLRR is an Lck-dependent inhibitory receptor in lymphocytes.

## Materials and Methods

### Sequence Retrieval and Alignment

Protein sequences were obtained *via* JGI (https://gold.jgi.doe.gov/index), NCBI (https://www.ncbi.nlm.nih.gov/protein/), and LanceletDB (http://genome.bucm.edu.cn/lancelet/index.php) databases and subjected to BLASTP searches to predict authentic sequences because the *Branchiostoma* genome sequence was incomplete at the time of this study. Lck protein sequences from nine vertebrate species were retrieved from the NCBI database, and each sequence was then used for BLASTP searches in the LanceletDB and JGI databases to identify the BbLck sequence. Reciprocally, for verification, the putative BbLck sequence in the NCBI database was used to search for vertebrate Lck sequences using BLASTP. The cloned BbLcLRR cDNA contained 2097 bp and was submitted to the GenBank database with the accession number MW390887. The domain structure was predicted using the Simple Modular Architecture Research Tool (SMART) (http://smart.embl.de/).

### Plasmids and siRNAs

Human Lck (GenBank, NP_001036236.1) was amplified from Jurkat TAg cells cDNA and cloned into the pcDNA3.1-Flag (Invitrogen). The sequence of BbLck was cloned from Chinese amphioxus (Branchiostoma belcheri, China) gut cDNA. The point mutations Y539F, Y655F and Y690F were mutated using pfuUltra II DNA polymerase according to the manufacturer’s instructions (Stratagene, San Diego, CA, USA). The scrambled control siRNA, including siLck (5′-CCACCCACAUGUGACACAU-3′) were synthesized by RiboBio Co. Ltd (Guangzhou, China).

### Antibodies and Reagents

Antibodies to Myc (9E10), actin (C4), Lck (SC-433), Lck phosphorylated at Tyr394 (SC-101728), LAT (FL-233), GAPDH(SC-25778), Lck inhibitor [7-Cyclopentyl-5-(4-phenoxyphenyl)-7H-pyrrolo(2,3d) pyrimidin-4-ylamine, SC-204052] were from Santa Cruz Biotechnology. Antibodies to LAT phosphorylated at Tyr132 (ab4476) and SLP76 phosphorylated at Tyr145 (ab75829) were from abcam. M2 antibody to Flag (F3165) was from Sigma-Aldrich (St Louis, MO, USA). Antibody specific for ERK phosphorylated at Thr202 and Tyr204 (E10) and phosphotyrosine specific mAb (pY100) were from Cell Signaling Technology. Anti-human CD3 (UCHT1) and PLC-γ1 phosphorylated at Tyr783 (612464) were from BD Pharmingen. Goat anti-mouse IgG (31160) was from Thermo Fisher Scientific. Horseradish peroxidase-conjugated secondary antibodies were from Jackson ImmunoResearch. IL-2 ELISA Ready-SET-Go (88–7025–88) was from eBioscience. Alexa Fluor 488-coupled chicken anti-mouse (A-21200) was from Invitrogen.

### Animal Immune Procedures and Antibody Affinity Purification

To produce a large amount of antibody and facilitate the affinity purification of antibody, we selected the New Zealand rabbit as the antigen immune host. The animal experiments were manipulated according to guidelines approved by the Animal Care and Ethics committee of Sun Yat-Sen University. The animal immune procedure was performed as previously described (26). Briefly, two rabbits were first intraperitoneally injected with the antigen protein mixed with an equal volume of Freund’s Complete Adjuvant (CFA, Sigma). After an interval of 14 days, the rabbit was injected a second time with the antigen protein mixed with Incomplete Freund’s Adjuvant (IFA, Sigma) and then immunized with the same method every week until the antibody titer met the needs of subsequent experiments.

We purified rabbit anti-BbLck serum with antigen-protein-coupled CNBr-activated Sepharose (GE Healthcare). The workflow is as follows: I. cDNA encoding BbLck was inserted into the pTwin-1 vector (New England Biolabs). The recombinant plasmid was transfected into DE3 (an *E. coli* strain) cells for protein expression. The recombinant protein was purified *via* binding to chitin beads and eluted with elution buffer containing 50 mM Tris-HCl (pH 6.0) and 500 mM NaCl. The recombinant protein was finally dissolved in CNBr Sepharose-compatible buffer containing NaHCO_3_ (pH 8.0) through buffer exchange by ultrafiltration (Millipore). The recombinant protein concentration was measured using the BCA and Bradford assays (Bio-Rad). II. Recombinant protein (1 mg/mL) was incubated with CNBr-activated Sepharose (GE Healthcare) at 4°C overnight. The rabbit anti-BbLck antiserum was purified according to the manufacturer’s instructions (GE Healthcare). Through this affinity purification procedure, we obtained antibodies that could specifically detect endogenous BbLck protein.

### Flow Cytometry Analysis

HEK293T cells were grown in a monolayer in 6-well plates one night before the experiment. After reaching an approximately 90% density, the cells were transfected with Flag vector or Flag-BbLcLRR vector. Twenty-four hours after transfection, the cells were collected and resuspended in 1 × phosphate-buffered saline (PBS) plus 1% BSA (w/v) and incubated with anti-Flag (M2) antibody (Sigma) for 1 h at 20-25°C. After three washes with resuspended buffer, the cells were stained with Alexa Fluor 488-labeled chicken anti-mouse IgG for 1 h at 20-25°C, followed by three washes with resuspended buffer. Cells were analyzed using a FACSCalibur flow cytometer (BD Biosciences) and CellQuest software. FlowJo software was used for data analysis.

### Cell Culture, Transfection, and Stimulation

Human leukemia Jurkat TAg cell line was cultured in RPMI1640 medium (Hyclone) supplemented with 10% (vol/vol) fetal bovine serum (Gibco), 100 U/ml streptomycin, and 100 U/ml penicillin (Sigma) at 37°C, 5% CO_2_. Cells in a logarithmic growth phase were transfected with indicated plasmid by nucleofection (Lonza 4D Nucleofector™ system). In each experiment, cells were transfected with the same total amount of DNA by the addition of the requisite quantity of empty vector. After transfection, cells were incubated in RPMI medium containing 10% FBS without penicillin and streptomycin for 48 h. For stimulation with antibody in Jurkat T cells, cells were washed with serum-free RPMI1640 medium, and stimulated with 10 μg/ml anti-CD3, which were crosslinked with goat anti-mouse IgG (10 μg/ml). HEK293T cells were cultured in DMEM (Hyclone) containing 10% FBS, penicillin and streptomycin. Transfections were carried out with PEI (Sigma-Aldrich).

### Western Blot Analysis and Coimmunoprecipitation

Cells were harvested and lysed in lysis buffer containing 20 mM Tris-HCl (pH 7.5), 150 mM NaCl, 5 mM EDTA (pH 8.0), 5 mM NaPPi, 1 mM sodium orthovanadate (Na_3_VO_4_), 1 mM PMSF, 1% Nonidet P-40, 10 μg/mL aprotinin, and 10 μg/mL leupeptin. After centrifugation at 13200 × *g* at 4°C to remove cellular debris, the supernatant was incubated with the indicated antibody overnight at 4°C, and the proteins were immunoprecipitated with protein G-Sepharose (GE Healthcare) for an additional 4 h at 4°C. The precipitated proteins or total cell lysates were separated by SDS-PAGE, and proteins were transferred to polyvinylidene fluoride (PVDF) membranes at 400 mA. The membranes were blocked with 4% BSA (dissolved in TBST buffer) and then incubated with the indicated primary antibody overnight at 4°C, followed by incubation with HRP-conjugated secondary antibodies for 1 h at room temperature. Immunoblots were developed using an X-ray film or with a ChemiDoc instrument (Bio-Rad).

### Immunofluorescence and Microscopy

Jurkat TAg T cells or amphioxus gut cells were fixed in 4% paraformaldehyde (PFA), layered onto poly-L-lysine (PLL)-coated microscope slides and permeabilized with PBS and Tween 0.1%. After blocking with 2% BSA in PBS for 30 min, cells were stained overnight at 4°C with the indicated primary antibodies. Cells were washed with PBS three times, after which fluorescent dye-conjugated secondary antibodies were added and incubated for 1 h at room temperature for double staining. Nuclei were counterstained with DAPI (1 μg/mL). As a negative control, secondary antibodies were used alone. Images were acquired using a FluoView FV1000 confocal microscope (Olympus) with an oil immersion objective (60× 1.4 NA, Plan-Apochromat; Olympus) using laser excitation at 405, 488, and 543 nm.

### Fish Immune Stimulation and Tissue Extraction

Amphioxi were obtained from Zhan Jiang, China, and fed algae every day at 20-22°C before being starved for two days before the experiments. Fish immune stimulation was performed as previously described (27). In brief, the amphioxi were sedated with MS-222 (20 mg/L, Sigma) for coelom injection with 15 μL of 0.5× PBS containing 10^5^ live *E. coli*, 10^5^ mouse erythrocytes, 15 μg of phytohemagglutinin (PHA) and 20 μg of pokeweed mitogen (Sigma). After stimulation for the indicated durations, amphioxus tissue separation was performed as previously described (28). Briefly, the work was performed using an ophthalmic scalpel and forceps with the help of a stereoscope (Olympus, SZ61TR). For gut primary cell extraction, gut tissue was cut into several segments and cells extracted with Dounce grinder. The cell strainer (BD-40 μm) was used to further remove tissue blocks.

### Mass Spectrometry

Protein samples were prepared for mass spectrometry measurements as previously described (29). In short, the indicated protein complex was subjected to SDS-PAGE. After the sample became a compact slit-like band after loading, the protein band was excised from a Coomassie-stained SDS-PAGE gel and fragmented into small pieces. Thereafter, the proteins were reduced with 10 mM dithiothreitol (DTT) for 30 min at 55°C, followed by alkylation with 55 mM iodoacetamide (IAA) for 30 min at room temperature in the dark. Overnight digestion at 37°C was performed with 0.6 μg of trypsin (Proteomics-grade, Promega) in 25 mM NH_4_HCO_3_. The resulting peptides were acidified with 0.1% formic acid (FA) to stop trypsin digestion (30).

### Luciferase Reporter Assay

For reporter assays, by using the Lonza 4D Nucleofector™ system, Jurkat TAg T cells were electroporated in triplicate with a combination of NF-κB and Renilla luciferase reporter plasmids, and the indicated plasmids. After 24 h, cells were treated with or without anti-CD3 for 8 h, lysed, and collected for the luciferase reporter assay. The luminescence was evaluated with a luciferase assay kit (Promega) using a Lumat LB 9507 tube luminometer (Berthold Technologies). Renilla luciferase reporter plasmids were used as internal controls.

### Measurement of IL-2 Production by ELISA

Jurkat TAg cells were stimulated for 24 h with anti-CD3 and the concentration of IL-2 in culture supernatants was determined by enzyme-linked immunosorbent assay (ELISA) according to the manufacturer’s instructions (eBioscience). A 96-well plate (Corning Costar) was coated overnight at 4°C with a monoclonal antibody against IL-2. Triplicates of IL-2 standards and supernatants from the cultured cells were then added to the plate and incubated for 2 h at room temperature. A biotinylated polyclonal antibody specific for IL-2 was added, followed by incubation for 1 h at room temperature, and then Avidin-HRP was added, followed by incubation for 30 min at room temperature. The amount of bound avidin was then assessed with 3,3’,5,5’-tetramethylbenzidine (TMB) peroxidase that was acidified with 2 N H_2_SO_4_. The absorbance of each well was measured at 450 and 570 nm using a spectrophotometric plate reader (BioTek Instruments).

### Quantitative Real-Time PCR

Quantitative real-time PCR was performed in a total volume of 10 μl with 5 μl of 2× realstar green power mixture (Genstar), 0.2 μl of primers, 0.5 μl of cDNA templates and double-distilled water. Real-time PCR was performed in LightCycler^®^ 480 System (Roche). Data were collected and quantified using the 2-ΔΔCt method according to the Ct values of target genes and normalized to endogenous control 18S RNA.

### Race PCR

Total RNA derived from amphioxus gut cells was used for Race PCR (GeneRacer kit, Invitrogen). Briefly, to obtain Race-ready RNA, aliquot amounts of RNA were dephosphorylated with CIP (Calf intestinal phosphatase) followed by decapping with TAP (Tobacco Acid Pyrophosphatase). And then, decapped RNA was ligated with RNA Oligo by T4 RNA ligase. Race-ready RNA was then reverse transcribed to cDNA with appropriate reverse transcriptase. Finally, these cDNAs were stored at -80°C.

### Statistical Analysis

Statistical Analysis was performed with a two-tailed unpaired *t*-test. *P*-values of < 0.05 were considered statistically significant. GraphPad 7.0 software and ImageJ software were used for graphs and statistical analysis.

## Results

### Cloning and Sequence Analyses of BbLck

**Figure 1:**
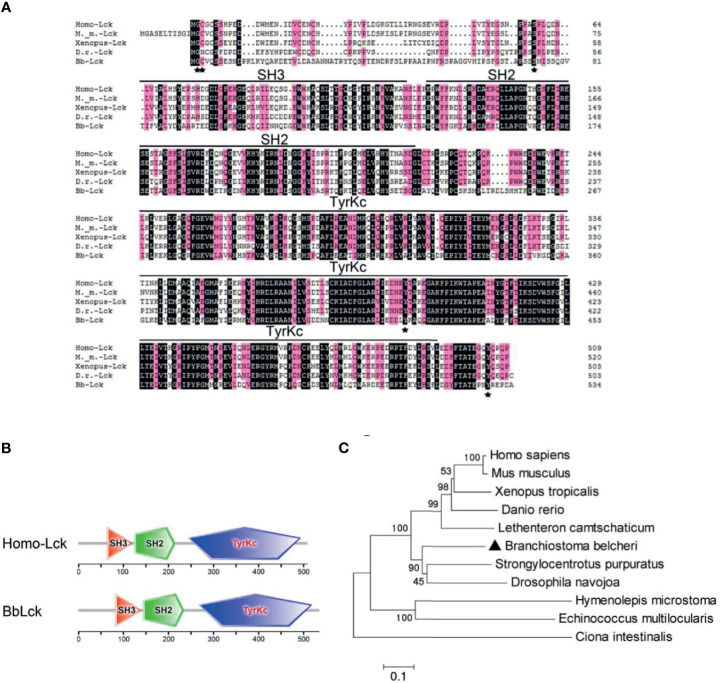
The homology and phylogenetic analysis of Lck derived from different species. **(A)** The similarities between Lck protein sequences determined by Clustal 8.0. **(B)** Domain comparison of Homo-Lck and BbLck using the sequence analysis tool SMART. **(C)** Phylogenetic tree of Lck sequences. T neighbor-joining phylogenetic tree was constructed by Mega5.0 on the basis of eleven different Lck sequences from GenBank. The accession numbers of the Lck sequences used in **(A–C)** are as follows: *Homo sapiens* Lck (GenBank, NP_001036236.1), *Mus musculus* Lck (GenBank, NP_001155904.1), *Xenopus tropicalis*Lck (GenBank, XP_012812855.2), *Danio rerio* Lck (GenBank, NP_001001596.1), *Ciona intestinalis* Lck (GenBank, XP_002123919.1),*Strongylocentrotus purpuratus* Lck (GenBank, NP_999783.1), *Drosophila navojoa* Lck (GenBank, XP_017960855.1), *Hymenolepis microstoma* Lck (GenBank, CDS29567.1), *Echinococcus multilocularis* Lck (GenBank, CUT99347.1); and *Lethenteron camtschaticum* Lck (GenBank, AIN44440.1).

To determine whether the Lck gene is present in the amphioxus, NCBI, JGI, and LanceletDB databases were used for BbLck sequence retrieval. Based on the retrieved gene sequences, we cloned the BbLck gene from *Branchiostoma belcheri* (Bb), and the deduced protein sequences are shown in the supplementary data (**Supplementary Figure 1**). BbLck encodes a 535-amino acid polypeptide with a highly conserved protein structure consisting of an SH3 domain, an SH2 domain adjacent to the SH3 domain, and a central tyrosine kinase (TyrKc) domain. Homology analysis of the BbLck protein sequence with other known Lck sequences, showed that its kinase domain and key amino acids corresponding to Gly 2 (myristoylation site), Cys 3 (palmitoylation site), Ser 59 (phosphorylated by MAPK), Y394 (activating phosphorylation site), and Y505 (inhibitory phosphorylation site) in human Lck are strongly conserved, which correlates with its highly conserved membrane localization, protein binding, kinase activation, and kinase activity inhibition (**Figure 1A**) (31–33). Using the SMART domain prediction analysis, the protein domains of BbLck were the same as those of the human Lck protein (**Figure 1B**). A phylogenetic tree based on the human, mouse, xenopus, zebrafish, lamprey, sea urchin, sea squirt, cestode, fly, and amphioxus Lck sequences was constructed. Phylogenetic analysis showed that BbLck clusters in invertebrates, in agreement with traditional taxonomy, and that BbLck is the closest ortholog to vertebrate Lck (**Figure 1C**).

### Characterization of BbLck Expression in Amphioxus

**Figure 2:**
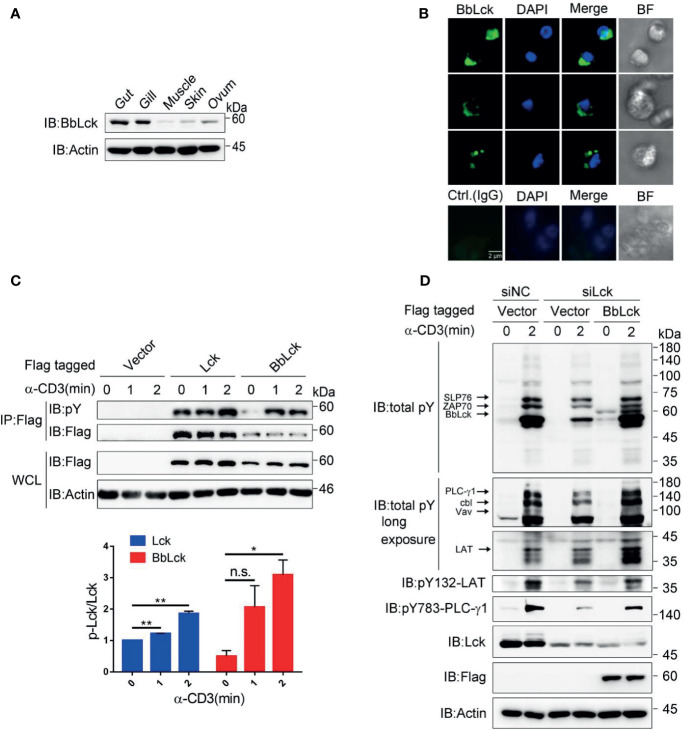
Characterization of BbLck distribution in amphioxus and function in Jukart TAg cells. **(A)** Different tissue distributions of BbLck. Homemade rabbit anti-BbLck antibody was used for immunoblotting. **(B)** Localization of BbLck in the gut cells of an amphioxus. Single cells were separated from gut tissue and then immunostained with BbLck antibody followed by fluorophore-conjugated secondary antibody (green). Nuclei were stained with DAPI dye (blue). Images were acquired using fluorescence microscopy at 400× magnification (scale bar, 2 μm). **(C)** Tyrosine phosphorylation of human Lck and BbLck. The indicated plasmids were transfected into Jurkat TAg cells for 48 h, and the cells were then stimulated with anti-CD3 for the indicated times. The identity of activated Lck was confirmed by anti-Flag immunoprecipitation followed by pY antibody immunoblotting. The densitometric quantification of the ratio of phosphorylated Lck to immunoprecipitated Lck (the ratio for the 0 min was set as 1) is shown on the bottom. **(D)** Jurkat TAg T cells were cotransfected with the siRNA designed specifically targeting Lck mRNA (siLck) and plasmid for 48 h, followed by stimulation with anti-CD3 for the indicated time. Whole-cell lysates (WCLs) were immunoblotted with pY antibody and the indicated antibodies. n.s., not significant; *P < 0.05, **P < 0.01 (two-tailed unpaired *t*-test). The data are presented as the mean (± s.e.m.). Data are representatives of three **(A, B, D)** or two **(C)** independent experiments.

To rule out the possibility that we cloned a BbLck pseudogene and to analyze the endogenous cellular expression of BbLck and its function. First, a specific homemade rabbit polyclonal antibody recognizing BbLck was prepared (**Supplementary Figures 2A–C**). The BbLck antibody could not recognize human Lck, while human Lck antibody did weakly recognize BbLck (**Supplementary Figure 2D**), suggesting they have species specificity. Using this antibody, we found that BbLck was expressed in the amphioxus immune organs (the gut and gill) (**Figure 2A**). Morphological observations showed lymphocyte-like cells in the gut organs of the amphioxi (28). Consistent with this finding, immunostaining for BbLck showed that BbLck proteins were localized in the cytosol of gut cells (**Figure 2B**).

### BbLck Is Functionally Homologous to Human Lck

In T cells, TCR ligation results in phosphorylation and activation of Lck, which in turn phosphorylates TCR subunits and triggers a cascade of tyrosine phosphorylations that leads to T-cell activation (12, 34). To demonstrate the tyrosine phosphorylation of BbLck, we transfected Jurkat TAg T cells with BbLck. Similar to human Lck, BbLck is tyrosine phosphorylated upon TCR stimulation (**Figure 2C**). BbLck partially rescued the decrease of tyrosine phosphorylation of TCR signaling proteins, such as SLP76, ZAP70, LAT, PLC-γ1, Vav and Cbl, induced by the knockdown of endogenous Lck expression in Jurkat TAg T cells (**Figure 2D**). Together, these results showed that BbLck was functionally homologous to human Lck in kinase function.

### BbLcLRR Cloning and Identification

**Figure 3:**
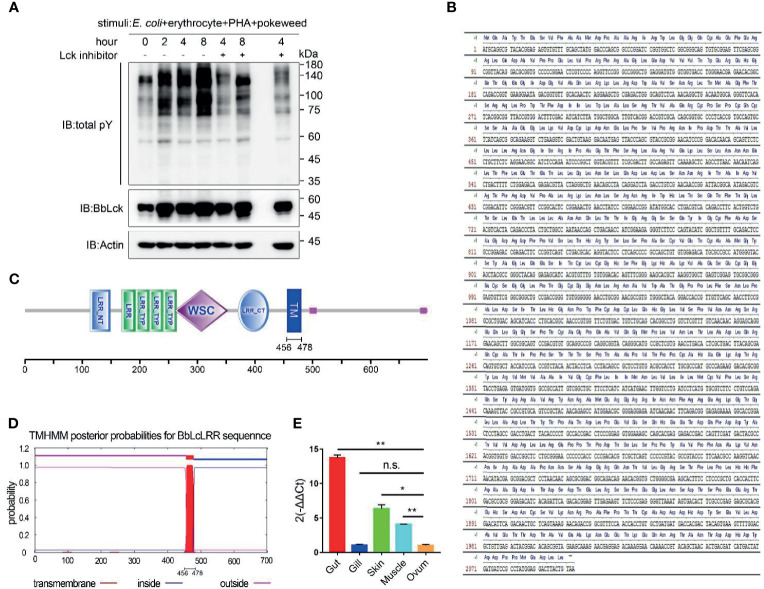
Identification of BbLcLRR. **(A)** Tyrosine phosphorylation of gut cellular proteins costimulated by *E coli* (10^5^), erythrocytes (10^5^), PHA (15 μg) and pokeweed (20 μg). Amphioxi were stimulated for the indicated duration in the absence or presence of Lck inhibitor, after which their gut cells were extracted. Tyrosine phosphorylation in whole-cell lysates was detected using the antibody P-Tyr-100. **(B)** Nucleotide sequence and deduced amino acid sequence of BbLcLRR. **(C)** Conserved domains prediction of BbLcLRR by SMART prediction tool online. **(D)** Transmembrane motif of BbLcLRR predicted by TMHMM server 2.0. **(E)** Relative expression level of BbLcLRR gene in various tissues of *Branchiostoma belcheri* by qRT-PCR. 2-ΔΔCt values (normalized to endogenous control 18S) represented the expression of the BbLcLRR gene in various tissues (gut, gill, muscle, skin, ovum) of *Branchiostoma belcheri*. n.s., not significant; *P < 0.05, **P < 0.01 (two-tailed unpaired *t*-test). The data are presented as the mean (± s.e.m.). Data are representatives of three **(A)** or two **(E)** independent experiments.

To explore the potential immune receptor binding to BbLck in amphioxus, we first analyzed whether tyrosine phosphorylation occurs in cephalochordates in response to immune stimulation which has not been documented. Therefore, amphioxi were injected with a cocktail containing PHA, pokeweed, and mouse erythrocytes to induce an immune response, as previously described (3, 27). We found that whole-cell tyrosine phosphorylation in the gut cells of amphioxi was substantially enhanced upon stimulation with this cocktail; while Lck inhibitor was coinjected, the induced tyrosine phosphorylation was obviously decreased (**Figure 3A**). These results implied the involvement of tyrosine phosphorylation of gut cellular proteins in amphioxus immune responses and that was largely dependent on BbLck activation, suggesting an important role of BbLck in amphioxus immune responses. We surmised that upon cocktail stimulation, BbLck could interact with immune receptors and other kinases or adaptor proteins to induce signaling cascade responses in the gut cells of the amphioxi. Using liquid chromatography-mass spectrometry (LC-MS) technology, the protein complexes immunoprecipitated with the homemade anti-BbLck antibody were analyzed. Intriguingly, one transmembrane protein containing leucine-rich repeats (that we named BbLcLRR) was found to interact with BbLck at the 8 h time point. Using the 5ʹ and 3ʹ race PCR, we cloned the full-length cDNA of BbLcLRR (submitted to GenBank, accession number MW390887). The coding sequence and deduced amino acid sequence of BbLcLRR was shown in **Figure 3B**, which shows that BbLcLRR consists of 699 amino acids with a predicted molecular weight of approximately 77 kDa. Domains predicted by the SMART program (http://smart.embl-heidelberg.de/) showed that the BbLcLRR contained LRR NT (N-terminal domain), followed by one LRR and three LRR TYPs (typical subfamily), WSC domain (putative carbohydrate binding domain), LRR CT (C-terminal domain), and TM (transmembrane region) (**Figure 3C**). The transmembrane region of BbLcLRR was also predicted using the TMHMM 2.0 server (**Figure 3D**). We further analyzed the expression of BbLcLRR in different amphioxus tissues with real-time PCR (**Figure 3E**). The result showed that BbLcLRR mRNA was highly expressed in gut cells as expected, but it had a relative lower mRNA level in gill cells (**Figure 3E**). The result seems not to be completely consistent with the tissue distribution of BbLck protein (**Figure 2A**), however, mRNA expression levels do not necessarily reflect the protein expression levels.

### Membrane Localization of BbLcLRR

**Figure 4:**
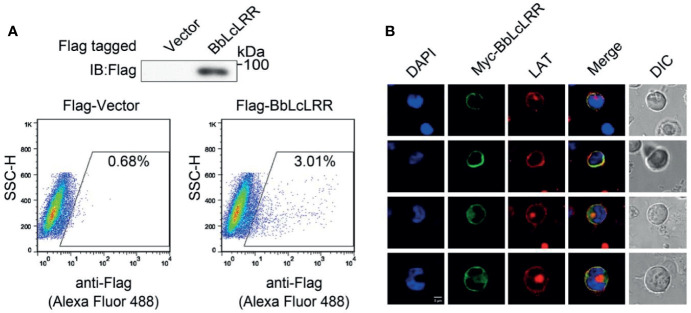
Membrane localization of BbLcLRR. **(A)** The Flag-BbLcLRR was identified by flow cytometry. Flag-BbLcLRR was overexpressed in HEK293T cells and stained with anti-Flag antibody followed by fluorophore-conjugated secondary antibody without cell membrane permeation treatment. **(B)** Cell membrane colocalization of overexpressed Myc-BbLcLRR (green) and endogenous LAT (red) in Jurkat TAg T cells. Nuclei were stained blue. Scale bar, 5 μm. Data are representatives at least three independent experiments.

Both prediction tools predicted that BbLcLRR has a transmembrane region from 456 to 478 aa, and the N-terminus has an extracellular localization. To confirm these predictions, we overexpressed N-terminal Flag-tagged BbLcLRR in HEK293T cells and immunostained it with anti-Flag antibody without cell permeabilization treatment, and analyzed afterwards by flow cytometry. The results showed that the Flag-tagged BbLcLRR could be stained without permeabilization, indicating that the N-terminus of BbLcLRR was localized outside of the cell membrane (**Figure 4A**). Furthermore, the colocalization of overexpressed BbLcLRR with the membrane-localized protein LAT (endogenous) could be detected in Jurkat TAg T cells (**Figure 4B**). Together, these results showed that BbLcLRR is a transmembrane protein.

### TCR Induces Tyrosine Phosphorylation of BbLcLRR *via* Lck

**Figure 5:**
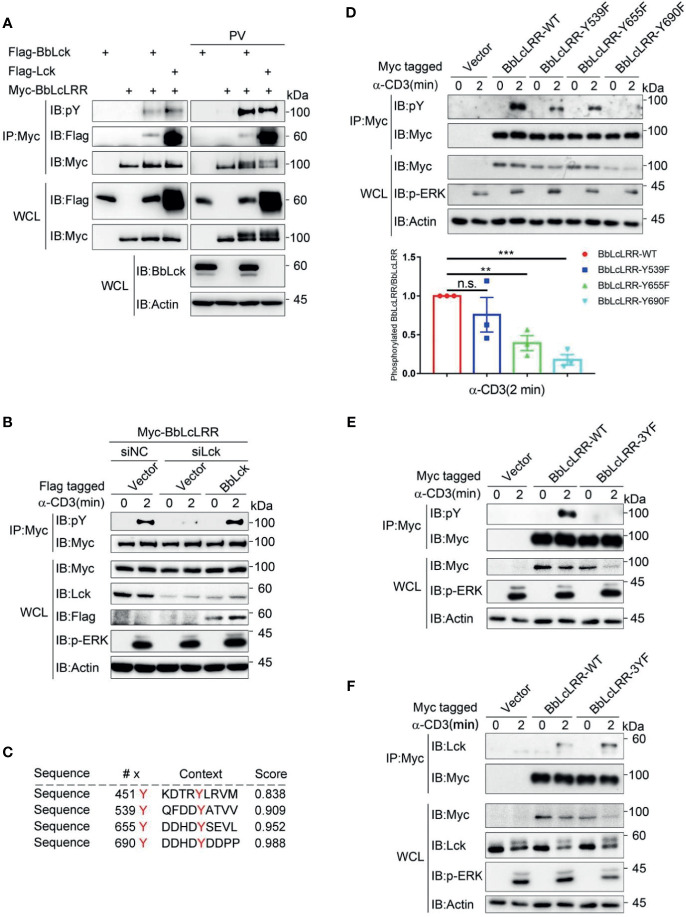
Lck phosphorylates BbLcLRR at multi-tyrosine sites. **(A)** Both human and amphioxus Lck bind to and phosphorylate BbLcLRR. The indicated plasmids were cotransfected into HEK293T cells, and cell lysates were immunoprecipitated with anti-Myc antibody followed by immunoblotting with the indicated antibodies. PV (pervanadate, phosphatase inhibitors) was used here for detection of tyrosine phosphorylation easily. **(B)** Knockdown of endogenous Lck reduced TCR-induced BbLcLRR tyrosine phosphorylation, and overexpression of BbLck rescued this reduction. The indicated siRNA and plasmids were cotransfected into Jurkat TAg T cells. After 48 h, the cells were collected and stimulated with anti-CD3 antibody for the indicated times. Cell lysates were immunoprecipitated by anti-Myc antibody, followed by immunoblotting with pY antibody (P-Tyr-100). **(C)** The tyrosine phosphorylation sites of BbLcLRR were predicted by the NetPhos 2.0 server. **(D)** The Y539F, Y655F and Y690F mutations decreased TCR-induced tyrosine phosphorylation of BbLcLRR. Cells were transfected with the indicated plasmids. After 48 h of transfection, the cells were treated as in **(B)**. The densitometric quantification of the ratio of phosphorylated BbLcLRR to immunoprecipitated BbLcLRR-WT or YF mutants (the ratio for the BbLcLRR-WT was set as 1) is shown on the bottom. **(E)** The simultaneous Y539F, Y655F and Y690F mutations (3YF) eliminated TCR-induced tyrosine phosphorylation of BbLcLRR. Cells were treated in the same way as in **(B)**. **(F)** BbLcLRR and BbLcLRR-3YF interacted with endogenous Lck with different affinities. Cells were treated in the same way as in **(B)**. Cell lysates were immunoprecipitated by anti-Myc antibody, followed by immunoblotting with anti-Lck antibody. n.s., not significant; **P < 0.01, and ***P < 0.001 (two-tailed unpaired *t*-test). The data are presented as the mean (± s.e.m.). Data are representatives of three **(B, D, E, F)** or two **(A)** independent experiments.

Because BbLcLRR was identified in the BbLck coimmunoprecipitates, we determined whether BbLcLRR directly interacts with and is phosphorylated by BbLck. We found that both BbLck and human Lck associated with BbLcLRR when coexpressed in HEK293T cells (**Figure 5A** and **Supplementary Figure 3**). And BbLcLRR was tyrosine phosphorylated when coexpressed with human Lck or BbLck, further treatment with pervanadate (PV, an inhibitor of protein-tyrosine phosphatases) enhanced the phosphorylation, meanwhile, a upshift band was observed for Myc-BbLcLRR (**Figure 5A**). These results indicated both human Lck and BbLck could phosphorylate BbLcLRR. Next, we tested whether BbLck or endogenous human Lck could phosphorylate BbLcLRR in T cells. As expected, TCR stimulation induced BbLcLRR tyrosine phosphorylation, which was inhibited by knockdown of endogenous Lck with siRNA, and this inhibition was rescued by co-expression of BbLck (**Figure 5B**). This result indicates that BbLck or endogenous human Lck could phosphorylate BbLcLRR upon TCR stimulation, suggesting BbLck-BbLcLRR axis could be activated in T cells. To map the tyrosine phosphorylation sites of BbLcLRR, we analyzed BbLcLRR sequence using the online NetPhos 2.0 server and found that four tyrosine residues of BbLcLRR, Y451, Y539, Y655, and Y690 are the sites with a high probability of phosphorylation (**Figure 5C**). We mutated Y539, Y655, and Y690 to phenylalanine (F) residues, respectively, and ruled out Y451 based on its predicted ectodomain localization (**Figures 3C, D**). By analyzing TCR-induced tyrosine phosphorylation of immunoprecipitated BbLcLRR, we found that each YF mutation decreased TCR-induced phosphorylation of BbLcLRR to various degrees (**Figure 5D**). In addition, simultaneous mutation of the three tyrosine sites to F residues (3YF) completely blocked TCR-induced phosphorylation of BbLcLRR (**Figure 5E**). Consistent with the association and dissociation kinetics of a kinase and its substrates that the binding of a kinase with its substrate is weaker than that with a pseudosubstrate (35), we observed that endogenous Lck associated with BbLcLRR-3YF was stronger than that with BbLcLRR wild-type in Jurkat TAg T cells (**Figure 5F**). Together, these data suggest that Lck is responsible for multi-tyrosine phosphorylation of the receptor BbLcLRR at its cytoplasmic tail.

### BbLcLRR Plays an Inhibitory Role on T Cell Activation Probably *via* Recruiting a SH2-Containing Phosphatase

**Figure 6:**
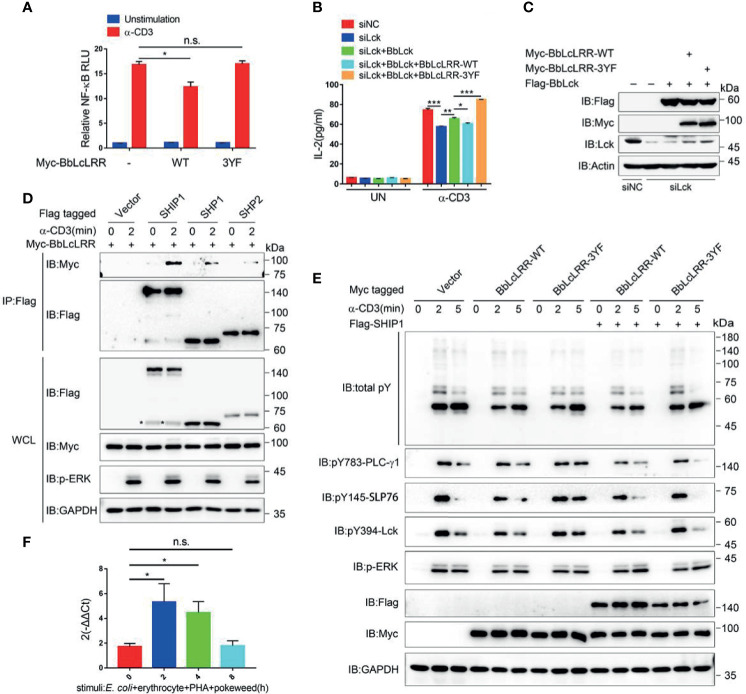
Function analysis of BbLcLRR in Jurkat TAg T cells. **(A)** Wild-type BbLcLRR, but not 3YF mutant BbLcLRR, inhibited CD3-induced NF-κB activity. 48 hours after cotransfection with the indicated plasmids, luciferase reporter vector driven by an NF-κB-responsive promoter and Renilla luciferase reporter, cells were stimulated with anti-CD3 antibody for 8 h, and relative NF-κB luciferase (NF-κB-Luc) activity was then measured (the NF-κB luciferase activity for vector control unstimulated was set as 1). **(B)** ELISA of IL-2 production in the culture supernatants of Jurkat TAg cells transfected with negative control (siNC) or siLck together with Flag-BbLck and Myc-BbLcLRR-WT or -3YF as indicated combination, followed by stimulation with anti-CD3 for 24h. **(C)** The protein expression levels in experiment of **(B)**. **(D)** BbLcLRR interaction with SHIP1, SHP1/2. The indicated plasmids were cotransfected into Jurkat TAg T cells. After 48 h, the cells were collected and stimulated with anti-CD3 antibody for the indicated times. Cell lysates were immunoprecipitated by anti-Flag antibody, followed by immunoblotting with indicated antibody. **(E)** Jurkat TAg T cells were transfected with empty vector, Myc-BbLcLRR-WT or BbLcLRR-3YF together with Flag-SHIP1 or not as indicated for 48 h, followed by stimulation with anti-CD3 for the indicated time. Whole-cell lysates (WCLs) were immunoblotted with the indicated antibodies. **(F)** Relative expression level of BbLcLRR gene of *Branchiostoma belcheri* stimulated with *E coli* (10^5^), erythrocytes (10^5^), PHA (15 μg) and pokeweed (20 μg) for various time by qRT-PCR. 2-ΔΔCt values (normalized to endogenous control 18S) represented the expression of the BbLcLRR gene in *Branchiostoma belcheri* stimulated various duration. n.s., not significant; *P < 0.05, **P < 0.01, and ***P < 0.001 (two-tailed unpaired *t*-test). The data are presented as the mean (± s.e.m.). Data are representative of at least three independent experiments.

The induced tyrosine phosphorylation of BbLcLRR by Lck raises the question as to whether BbLcLRR plays a role in T cell activation. Analysis of the sequences containing Y539 and Y655 showed that both DD**Y** (539)AT**V** and HD**Y** (655)SE**V** are conserved in the key tyrosine and valine positions with ITIM motif I/V/Lx**Y**xxL/**V** (where x represents any amino acid) (36), suggesting they are amphioxus ITIM motifs. The sequence containing Y690 is HDY (690)DDP, which is conserved with the reported amphioxus ITIM-like motif (AIYQSD) in amphioxus VCP, a member of the Ig superfamily (37). To verify whether BbLcLRR plays an inhibitory role as an ITIM-containing receptor, we transfected Jurkat TAg cells with BbLcLRR wild-type or BbLcLRR-3YF together with an NF-κB-luciferase reporter plasmid and then stimulated with anti-CD3 antibody. We found that BbLcLRR wild-type inhibited TCR-induced NF-κB activation significantly, whereas the BbLcLRR-3YF mutant had no effect on NF-κB-luc activity (**Figure 6A**). Further ELISA analysis of the effect of BbLcLRR wild-type and BbLcLRR-3YF on TCR-induced IL-2 production showed that, while knockdown of endogenous Lck inhibited IL-2 production, overexpression of BbLck could rescue this inhibition, overexpression of BbLcLRR wild-type, but not BbLcLRR-3YF, together with BbLck blocked this rescue (**Figures 6B, C**). These data suggest that BbLcLRR negatively regulates T cell activation which requires Lck/BbLck-mediated inhibitory phosphorylation on its ITIM or ITIM-like motifs.

ITIM-containing inhibitory receptors can recruit SH2-containing phosphatase and thus attenuate signaling in a broadly similar manner (38). Therefore, we analyzed whether BbLcLRR could interact with SH2-containing phosphatases, such as mammalian SHIP1, SHP1 and SHP2. The result showed that while BbLcLRR did not interact with the three phosphatases in resting T cells, it did bind to them with different degrees upon TCR stimulation (**Figure 6D**). We next investigated the impact of BbLcLRR on the phosphorylation of TCR signaling proteins. Due to the strong binding between BbLcLRR and SHIP1 (**Figure 6D**), we transfected Jurkat T cells with BbLcLRR wild-type or 3YF mutant alone or together with SHIP1, respectively. The results in **Figure 6E** showed that, BbLcLRR wild-type expression alone reduced TCR-induced tyrosine phosphorylation of PLC-γ1, SLP76 and Lck, and coexpression of SHIP1 with BbLcLRR wild-type further slightly strengthen the inhibition. Whereas compared with wild-type expression alone, BbLcLRR-3YF alone had much less inhibitory impact and even increased the phosphorylation, especially that of PLC-γ1, SLP76 and Lck, at 5 min stimulation (**Figure 6E**, lane 9), and the coexpression of 3YF mutant with SHIP1 showed similar phosphorylation at 2 min stimulation to 3YF alone as expected, the phosphorylation of PLC-γ1, SLP76 and Lck at 5 min stimulation was almost completely disappeared (**Figure 6E**, lane 15). Of note, the ERK activation level seemed not to be affected by different transfections (**Figure 6E**), which was unexpected because of the decreased phosphorylation of TCR proximal signaling proteins. We speculated that other signaling other than PLC-γ1 signaling may compensate the ERK activation, such as phosphatidylinositol-3-kinase (PI3K) could mediate ERK activation upon TCR stimulation (39, 40). As to the increased tyrosine phosphorylation of PLC-γ1, SLP76 and Lck at 5 min stimulation of BbLcLRR-3YF expression alone, it might be caused by the competition of overexpressed BbLcLRR-3YF with an phosphatases-associated endogenous inhibitory receptor and thus excluding these phosphatases from TCR signaling to sustain the tyrosine phosphorylation, which may also explain the 3YF mutant increasing IL-2 production in **Figure 6B**; as to the disappeared phosphorylation of PLC-γ1, SLP76 and Lck at 5 min stimulation of BbLcLRR-3YF coexpression with SHIP1, it might be caused by the overexpressed SHIP1(which should be excluded by the 3YF) binding to an induced endogenous inhibitory receptor after TCR stimulation for 5 min and then efficiently dephosphorylation of TCR signaling proteins. Also, considering the integration into TCR signalsome, BbLcLRR-WT-SHIP1 should be less efficient than SHIP1 with endogenous inhibitory receptor in T cells, therefore the phosphorylation of TCR signaling proteins at 5 min stimulation was decreased but not disappeared when coexpression of BbLcLRR wild-type with SHIP1.

Moreover, we searched the NCBI database and found that amphioxus BbINPPL1(Accession No. XP_019617039.1) is homologous to SHIP1, and Bb-PTPN11a (Accession No. XP_019637720.1) is homologous to SHP1/2 (**Supplementary Figure 4**). These results suggest BbLcLRR may exert its inhibitory function through similar mechanisms in amphioxus. Furthermore, we checked the mRNA expression phase of BbLcLRR in gut cells by qRT-PCR. We found that the mRNA expression level of BbLcLRR was relatively low in resting gut cells, and was significantly increased after stimulation with a cocktail of immunostimulants for two hours and back to baseline level after 8 hours stimulation (**Figure 6F**). This result indicated that BbLcLRR may be an activation-induced inhibitory receptor by recruiting SH2-containing phosphatases.

## Discussion

In this study, we first elucidated the amphioxus Lck which is structurally and functionally homologous to human Lck. We also discovered a novel Lck-associated receptor BbLcLRR and demonstrated that it is an inhibitory immunoreceptor that depends on Lck or BbLck-mediated inhibitory phosphorylation. Therefore, BbLck-regulated and BbLcLRR-mediated immune inhibitory pathways may exist in amphioxus.

In vertebrates, Lck is predominantly expressed in lymphocytes (8). We found that BbLck was majorly expressed in the gut and gill tissues of the amphioxi, which strongly suggests that lymphocyte-like cells and important adaptive immune response elements have emerged in gut or gill tissues of the amphioxi, consistent with the findings of the presence of lymphocyte-like cells in gills (28). Tyrosine phosphorylation of Lck and Lck-mediated tyrosine phosphorylation of receptors and signaling proteins are hallmarks of T-cell activation. Similar to human Lck, BbLck could be tyrosine phosphorylated upon TCR stimulation and partially replace human Lck functionally. Moreover, the cocktail of immunostimulants induced tyrosine phosphorylation of gut cellular proteins could be decreased by Lck specific inhibitor. Therefore, a similar signaling pathway mediated by BbLck may exist in amphioxus.

The inhibitory immunoreceptors could be mainly divided into two distinct functional categories: receptors that express constitutively in resting cells and modulate the signaling threshold for immune cell activation, and receptors which are induced by immune activation and mediate the negative feedback of immune cell activation (41). Based on the finding of BbLcLRR in BbLck immunoprecipitates from the gut cell lysates stimulated for 8 hours and the mRNA expression phase of BbLcLRR, we may conclude that BbLcLRR is an immune activation-induced ‘negative feedback’ receptor.

Intriguingly, the sequence (**T**R**Y**LR**V**) in the predicted ectodomain containing BbLcLRR Y451 is highly conserved with the immunoreceptor tyrosine-based switch motif (ITSM), defined as TxYxxV/I, which has been described in receptors conveying either an activating or inhibitory signal (42). Extracellular phosphorylation of a receptor mediated by extracellular kinases plays an important role in receptor function (43). Whether this ITSM of BbLcLRR is phosphorylated and how it functions deserve further studies.

Lck binds CD4 and CD8 coreceptors to initiate its activation upon stimulation and then phosphorylates the tyrosine on ITAMs of CD3 subunits of TCR complex to trigger TCR signaling, Lck can also phosphorylate the ITIMs of HLA-specific killer cell inhibitory receptors (KIR) in NK and T cells (14), and phosphorylate both the ITIM and ITSM of PD-1 in T cells (15).Dual phosphorylation of PD-1 by Lck is required for its optimal SHP2 interaction and thus its inhibitory function (15). The findings of ITIM-containing BbLcLRR and BbLck-mediated activation of BbLcLRR inhibitory function and that BbLcLRR inhibited T cell activation probably *via* recruiting a SH2-containing phosphatase in T cells imply the homologous inhibitory regulation mechanism and professional immune cell type might exist in amphioxus.

In summary, our study suggests the existence of a novel BbLck-regulated inhibitory immunoreceptor BbLcLRR pathway in amphioxus, implying a tolerance and homeostasis mechanism of amphioxus immune system and the evolutionary conservatism of Lck-regulated inhibitory receptor pathway.

## Data Availability Statement

The datasets presented in this study can be found in online repositories. The names of the repository/repositories and accession number(s) can be found in the article/**Supplementary Material**.

## Ethics Statement

The animal study was reviewed and approved by the Animal Care and Ethics committee of Sun Yat-Sen University.

## Author Contributions

YL conceived and supervised this study and wrote the manuscript. JZ, ZX, YZ, XQ, SM, CD, TZ and XL performed the experiments. JZ and ZX prepared the manuscript. ZX organized the figures. SH gave critical suggestions and amphioxus materials. All authors contributed to the article and approved the submitted version.

## Funding

This study was supported by the National Natural Science Foundation of China (31670893, 31370886), the Guangzhou Science and Technology Project (201904010445), Guangdong Basic and Applied Basic Research Foundation (2021A1515010543) and Guangdong Science and Technology Department (2020B1212060031). 

## Conflict of Interest

The authors declare that the research was conducted in the absence of any commercial or financial relationships that could be construed as a potential conflict of interest.

## Supplementary Material

Supplementary Figure 1 Amnio acid sequence of BbLck.

Supplementary Figure 2 Homemade BbLck polyclonal antibody was attained *via* rabbit immunization. **(A)** Prokaryotic BbLck was purified by CNBr Sepharose. **(B)** Homemade antisera efficacy and specificity were measured *via* western blot analysis. **(C)** Homemade antisera specificity improved after affinity purification: a, e, i. Flag-vector expressed in HEK293T cells. b, f, j: Flag-BbLck expressed in HEK293T cells. c, g, k: gut tissues were used for endogenous BbLck protein detection. d, h, i: gut cells were used for endogenous BbLck protein detection. **(D)** The species specificity of human Lck antibody (mouse monoclonal) and homemade BbLck rabbit polyclonal antibody. Human Lck antibody or BbLck antibody was used to blot the overexpressed human Lck and BbLck in HEK293T, and to blot the endogenous Lck in Jurkat cells.

Supplementary Figure 3 Association of BbLcLRR with human or amphioxus Lck. The indicated plasmids were cotransfected into HEK293T cells, and cell lysates were immunoprecipitated with anti-Flag antibody followed by immunoblotting with the indicated antibodies.

Supplementary Figure 4 Domain analysis of human SHIP1, SHP1, SHP2, and amphioxus Bb-INPPL1 (Accession No. XP_019617039.1), Bb-PTPN11a (Accession No. XP_019637720.1).

## References

[1] Alder MN Rogozin IB Iyer LM Glazko GV Cooper MD Pancer Z. Diversity and Function of Adaptive Immune Receptors in a Jawless Vertebrate. Science (2005) 310(5756):1970–3. 10.1126/science.1119420 16373579

[2] Rast JP Buckley KM. Lamprey Immunity is Far From Primitive. Proc Natl Acad Sci USA (2013) 110(15):5746–7. 10.1073/pnas.1303541110 PMC3625256 23553834

[3] Li J Das S Herrin BR Hirano M Cooper MD. Definition of a Third VLR Gene in Hagfish. Proc Natl Acad Sci USA (2013) 110(37):15013–8. 10.1073/pnas.1314540110 PMC3773805 23980174

[4] Yuan S Ruan J Huang S Chen S Xu A. Amphioxus as a Model for Investigating Evolution of the Vertebrate Immune System. Dev Comp Immunol (2015) 48(2):297–305. 10.1016/j.dci.2014.05.004 24877655

[5] Abi-Rached L Gilles A Shiina T Pontarotti P Inoko H. Evidence of En Bloc Duplication in Vertebrate Genomes. Nat Genet (2002) 31(1):100–5. 10.1038/ng855 11967531

[6] Huang S Tao X Yuan S Zhang Y Li P Beilinson HA. Discovery of an Active Rag Transposon Illuminates the Origins of V(D)J Recombination. Cell (2016) 166(1):102–14. 10.1016/j.cell.2016.05.032 PMC5017859 27293192

[7] Zhang Y Xu K Deng A Fu X Xu A Liu X. An Amphioxus RAG1-like DNA Fragment Encodes a Functional Central Domain of Vertebrate Core RAG1. Proc Natl Acad Sci USA (2014) 111(1):397–402. 10.1073/pnas.1318843111 24368847 PMC3890805

[8] Perlmutter RM Marth JD Lewis DB Peet R Ziegler SF Wilson CB. Structure and Expression of Lck Transcripts in Human Lymphoid Cells. J Cell Biochem (1988) 38(2):117–26. 10.1002/jcb.240380206 3265417

[9] Rudd CE Trevillyan JM Dasgupta JD Wong LL Schlossman SF. The CD4 Receptor is Complexed in Detergent Lysates to a Protein-Tyrosine Kinase (pp58) From Human T Lymphocytes. Proc Natl Acad Sci USA (1988) 85(14):5190–4. 10.1073/pnas.85.14.5190 PMC281714 2455897

[10] Veillette A Bookman MA Horak EM Bolen JB. The CD4 and CD8 T Cell Surface Antigens are Associated With the Internal Membrane Tyrosine-Protein Kinase P56lck. Cell (1988) 55(2):301–8. 10.1016/0092-8674(88)90053-0 3262426

[11] Gascoigne NR Casas J Brzostek J Rybakin V. Initiation of TCR Phosphorylation and Signal Transduction. Front Immunol (2011) 2:72. 10.3389/fimmu.2011.00072 22566861 PMC3342367

[12] Gaud G Lesourne R Love PE. Regulatory Mechanisms in T Cell Receptor Signalling. Nat Rev Immunol (2018) 18(8):485–97. 10.1038/s41577-018-0020-8 29789755

[13] Wang QL Liang JQ Gong BN Xie JJ Yi YT Lan X. T Cell Receptor (Tcr)-Induced PLC-gamma1 Sumoylation Via PIASxbeta and PIAS3 Sumo E3 Ligases Regulates the Microcluster Assembly and Physiological Function of PLC-Gamma1. Front Immunol (2019) 10:314. 10.3389/fimmu.2019.00314 30873169 PMC6403162

[14] Marti F Xu CW Selvakumar A Brent R Dupont B King PD. LCK-Phosphorylated Human Killer Cell-Inhibitory Receptors Recruit and Activate Phosphatidylinositol 3-Kinase. Proc Natl Acad Sci USA (1998) 95(20):11810–5. 10.1073/pnas.95.20.11810 PMC21722 9751747

[15] Hui E Cheung J Zhu J Su X Taylor MJ Wallweber HA. T Cell Costimulatory Receptor CD28 is a Primary Target for PD-1-mediated Inhibition. Science (2017) 355(6332):1428–33. 10.1126/science.aaf1292 PMC6286077 28280247

[16] Stirnweiss A Hartig R Gieseler S Lindquist JA Reichardt P Philipsen L. T Cell Activation Results in Conformational Changes in the Src Family Kinase Lck to Induce its Activation. Sci Signal (2013) 6(263):ra13. 10.1126/scisignal.2003607 23423439

[17] Rossy J Williamson DJ Gaus K. How Does the Kinase Lck Phosphorylate the T Cell Receptor? Spatial Organization as a Regulatory Mechanism. Front Immunol (2012) 3:167. 10.3389/fimmu.2012.00167 22723799 PMC3377954

[18] Xu W Harrison SC Eck MJ. Three-Dimensional Structure of the Tyrosine Kinase C-Src. Nature (1997) 385(6617):595–602. 10.1038/385595a0 9024657

[19] Hardwick JS Sefton BM. Activation of the Lck Tyrosine Protein Kinase by Hydrogen Peroxide Requires the Phosphorylation of Tyr-394. Proc Natl Acad Sci USA (1995) 92(10):4527–31. 10.1073/pnas.92.10.4527 PMC41977 7538674

[20] Hui E Vale RD. In Vitro Membrane Reconstitution of the T-cell Receptor Proximal Signaling Network. Nat Struct Mol Biol (2014) 21(2):133–42. 10.1038/nsmb.2762 PMC4062301 24463463

[21] D’Oro U Vacchio MS Weissman AM Ashwell JD. Activation of the Lck Tyrosine Kinase Targets Cell Surface T Cell Antigen Receptors for Lysosomal Degradation. Immunity (1997) 7(5):619–28. 10.1016/s1074-7613(00)80383-0 9390686

[22] Zikherman J Jenne C Watson S Doan K Raschke W Goodnow CC. Cd45-Csk Phosphatase-Kinase Titration Uncouples Basal and Inducible T Cell Receptor Signaling During Thymic Development. Immunity (2010) 32(3):342–54. 10.1016/j.immuni.2010.03.006 PMC2865198 20346773

[23] Bougeret C Delaunay T Romero F Jullien P Sabe H Hanafusa H. Detection of a Physical and Functional Interaction Between Csk and Lck Which Involves the SH2 Domain of Csk and is Mediated by Autophosphorylation of Lck on Tyrosine 394. J Biol Chem (1996) 271(13):7465–72. 10.1074/jbc.271.13.7465 8631775

[24] al-Ramadi BK Nakamura T Leitenberg D Bothwell AL. Deficient Expression of p56(lck) in Th2 Cells Leads to Partial TCR Signaling and a Dysregulation in Lymphokine mRNA Levels. J Immunol (1996) 157(11):4751–61. 8943376

[25] Straus DB Weiss A. Genetic Evidence for the Involvement of the Lck Tyrosine Kinase in Signal Transduction Through the T Cell Antigen Receptor. Cell (1992) 70(4):585–93. 10.1016/0092-8674(92)90428-f 1505025

[26] Schunk MK Macallum GE. Applications and Optimization of Immunization Procedures. ILAR J (2005) 46(3):241–57. 10.1093/ilar.46.3.241 15953832

[27] Pancer Z Amemiya CT Ehrhardt GR Ceitlin J Gartland GL Cooper MD. Somatic Diversification of Variable Lymphocyte Receptors in the Agnathan Sea Lamprey. Nature (2004) 430(6996):174–80. 10.1038/nature02740 15241406

[28] Huang G Xie X Han Y Fan L Chen J Mou C. The Identification of Lymphocyte-Like Cells and Lymphoid-Related Genes in Amphioxus Indicates the Twilight for the Emergence of Adaptive Immune System. PloS One (2007) 2(2):e206. 10.1371/journal.pone.0000206 17299586 PMC1784065

[29] Aebersold R Mann M. Mass Spectrometry-Based Proteomics. Nature (2003) 422(6928):198–207. 10.1038/nature01511 12634793

[30] Hoberg AM Otteneder M Marnett LJ Poulsen HE. Measurement of the Malondialdehyde-2’-Deoxyguanosine Adduct in Human Urine by Immuno-Extraction and Liquid Chromatography/Atmospheric Pressure Chemical Ionization Tandem Mass Spectrometry. J Mass Spectrom (2004) 39(1):38–42. 10.1002/jms.547 14760611

[31] McNeill L Salmond RJ Cooper JC Carret CK Cassady-Cain RL Roche-Molina M. The Differential Regulation of Lck Kinase Phosphorylation Sites by CD45 is Critical for T Cell Receptor Signaling Responses. Immunity (2007) 27(3):425–37. 10.1016/j.immuni.2007.07.015 17719247

[32] Park I Chung J Walsh CT Yun Y Strominger JL Shin J. Phosphotyrosine-Independent Binding of a 62-kDa Protein to the Src Homology 2 (SH2) Domain of p56lck and its Regulation by Phosphorylation of Ser-59 in the Lck Unique N-terminal Region. Proc Natl Acad Sci USA (1995) 92(26):12338–42. 10.1073/pnas.92.26.12338 PMC40352 8618896

[33] Dutta D Barr VA Akpan I Mittelstadt PR Singha LI Samelson LE. Recruitment of Calcineurin to the TCR Positively Regulates T Cell Activation. Nat Immunol (2017) 18(2):196–204. 10.1038/ni.3640 27941787 PMC6352896

[34] van Oers NS Killeen N Weiss A. Lck Regulates the Tyrosine Phosphorylation of the T Cell Receptor Subunits and ZAP-70 in Murine Thymocytes. J Exp Med (1996) 183(3):1053–62. 10.1084/jem.183.3.1053 PMC2192313 8642247

[35] Eldar-Finkelman H Eisenstein M. Peptide Inhibitors Targeting Protein Kinases. Curr Pharm Des (2009) 15(21):2463–70. 10.2174/138161209788682253 19601843

[36] Veillette A Latour S Davidson D. Negative Regulation of Immunoreceptor Signaling. Annu Rev Immunol (2002) 20:669–707. 10.1146/annurev.immunol.20.081501.130710 11861615

[37] Yu C Dong M Wu X Li S Huang S Su J. Genes “Waiting” for Recruitment by the Adaptive Immune System: The Insights From Amphioxus. J Immunol (2005) 174(6):3493–500. 10.4049/jimmunol.174.6.3493 15749885

[38] Abbas AK Lichtman AH Pillai S. Cellular and Molecular Immunology E-Book. 10 th. 1600 John F. Kennedy Blvd: Elsevier (2021). p. 175. Available at: https://books.google.com.

[39] Robertson LK Mireau LR Ostergaard HL. A Role for Phosphatidylinositol 3-Kinase in TCR-stimulated ERK Activation Leading to Paxillin Phosphorylation and CTL Degranulation. J Immunol (2005) 175(12):8138–45. 10.4049/jimmunol.175.12.8138 16339552

[40] Ladygina N Gottipati S Ngo K Castro G Ma JY Banie H. PI3Kgamma Kinase Activity is Required for Optimal T-cell Activation and Differentiation. Eur J Immunol (2013) 43(12):3183–96. 10.1002/eji.201343812 PMC4209804 24030559

[41] Rumpret M Drylewicz J Ackermans LJE Borghans JAM Medzhitov R Meyaard L. Functional Categories of Immune Inhibitory Receptors. Nat Rev Immunol (2020) 20(12):771–80. 10.1038/s41577-020-0352-z 32612208

[42] Boussiotis VA Chatterjee P Li L. Biochemical Signaling of PD-1 on T Cells and its Functional Implications. Cancer J (2014) 20(4):265–71. 10.1097/PPO.0000000000000059 PMC4151049 25098287

[43] Hanamura K Washburn HR Sheffler-Collins SI Xia NL Henderson N Tillu DV. Extracellular Phosphorylation of a Receptor Tyrosine Kinase Controls Synaptic Localization of NMDA Receptors and Regulates Pathological Pain. PloS Biol (2017) 15(7):e2002457. 10.1371/journal.pbio.2002457 28719605 PMC5515392

