# Supplementary figures and images for: Identification and Characterization of the Amphioxus Lck and Its Associated Tyrosine Phosphorylation-Dependent Inhibitory LRR Receptor
Source: Front Immunol. 2021 Jun 3;12:656366. doi: 10.3389/fimmu.2021.656366 (PMC8211107; doi:10.3389/fimmu.2021.656366)

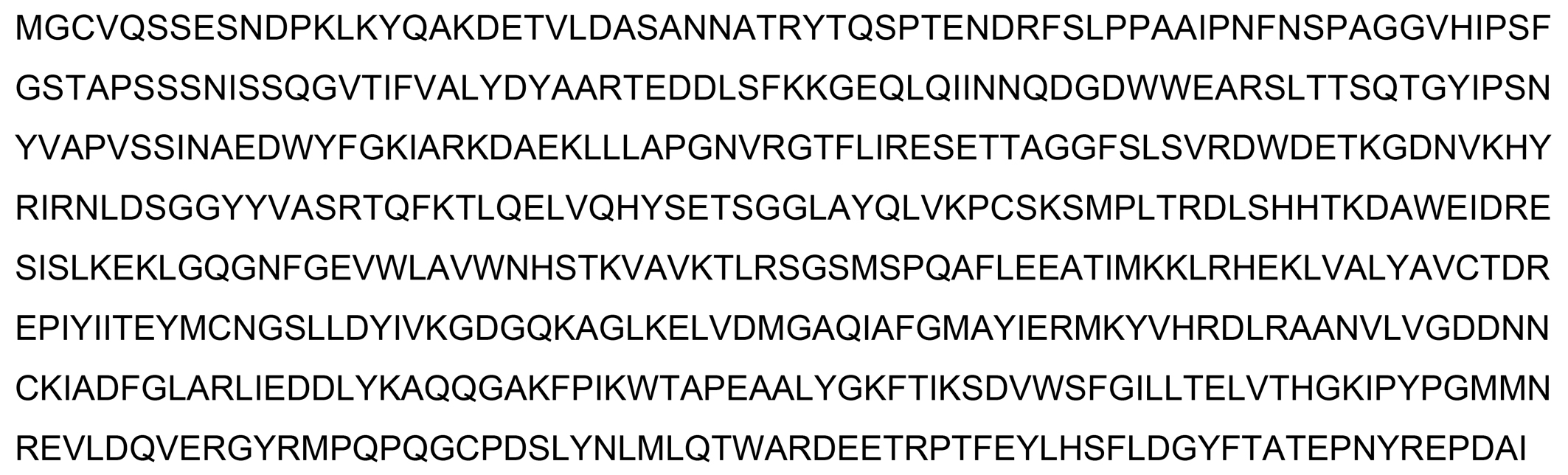

Supplement: Supplementary Figure 1 — Amnio acid sequence of BbLck. [file Image_1.jpeg]

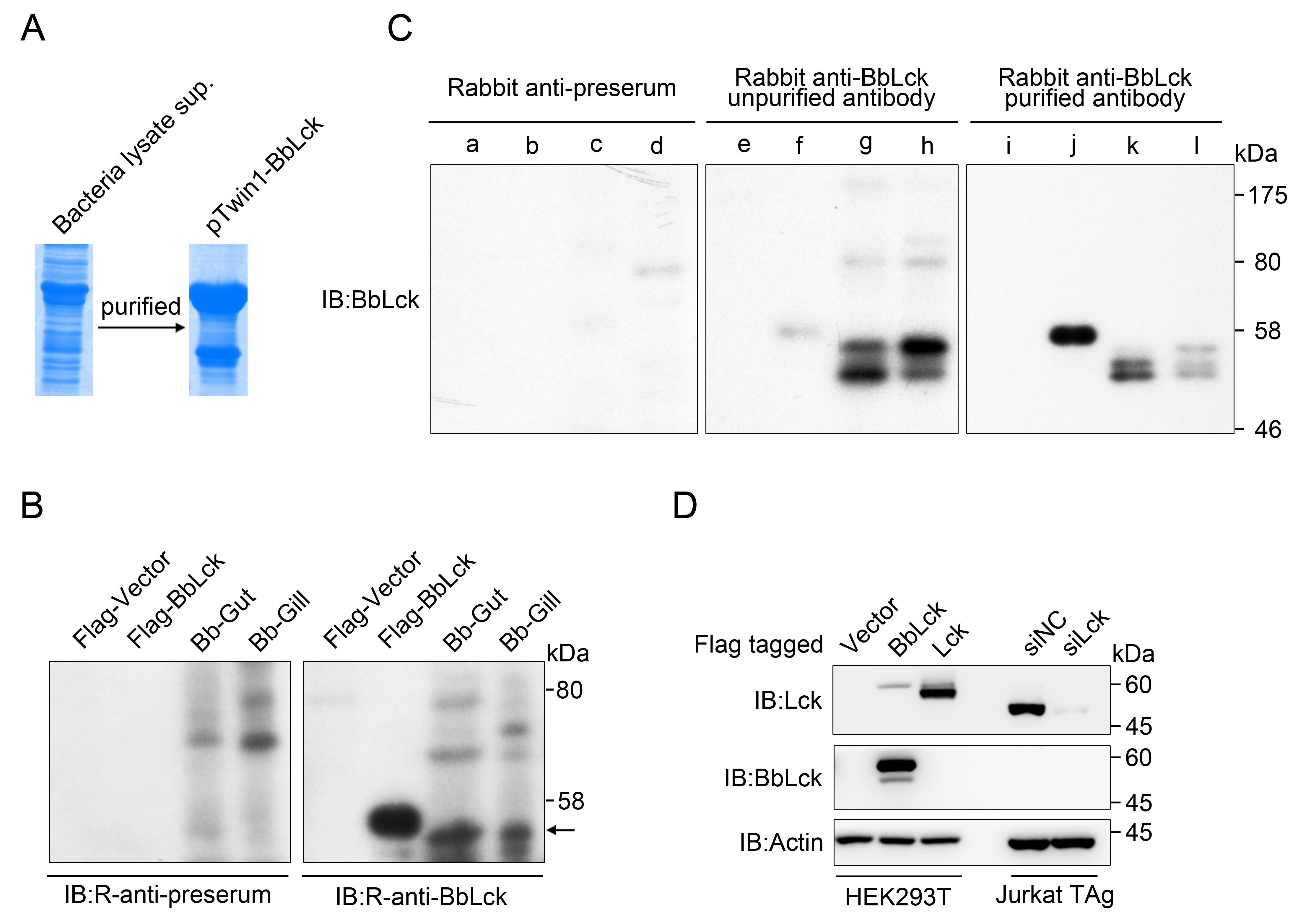

Supplement: Supplementary Figure 2 — Homemade BbLck polyclonal antibody was attained via rabbit immunization. (A) Prokaryotic BbLck was purified by CNBr Sepharose. (B) Homemade antisera efficacy and specificity were measured via western blot analysis. (C) Homemade antisera specificity improved after affinity purification: a, e, i. Flag-vector expressed in HEK293T cells. b, f, j: Flag-BbLck expressed in HEK293T cells. c, g, k: gut tissues were used for endogenous BbLck protein detection. d, h, i: gut cells were used for endogenous BbLck protein detection. (D) The species specificity of human Lck antibody (mouse monoclonal) and homemade BbLck rabbit polyclonal antibody. Human Lck antibody or BbLck antibody was used to blot the overexpressed human Lck and BbLck in HEK293T, and to blot the endogenous Lck in Jurkat cells. [file Image_2.jpg]

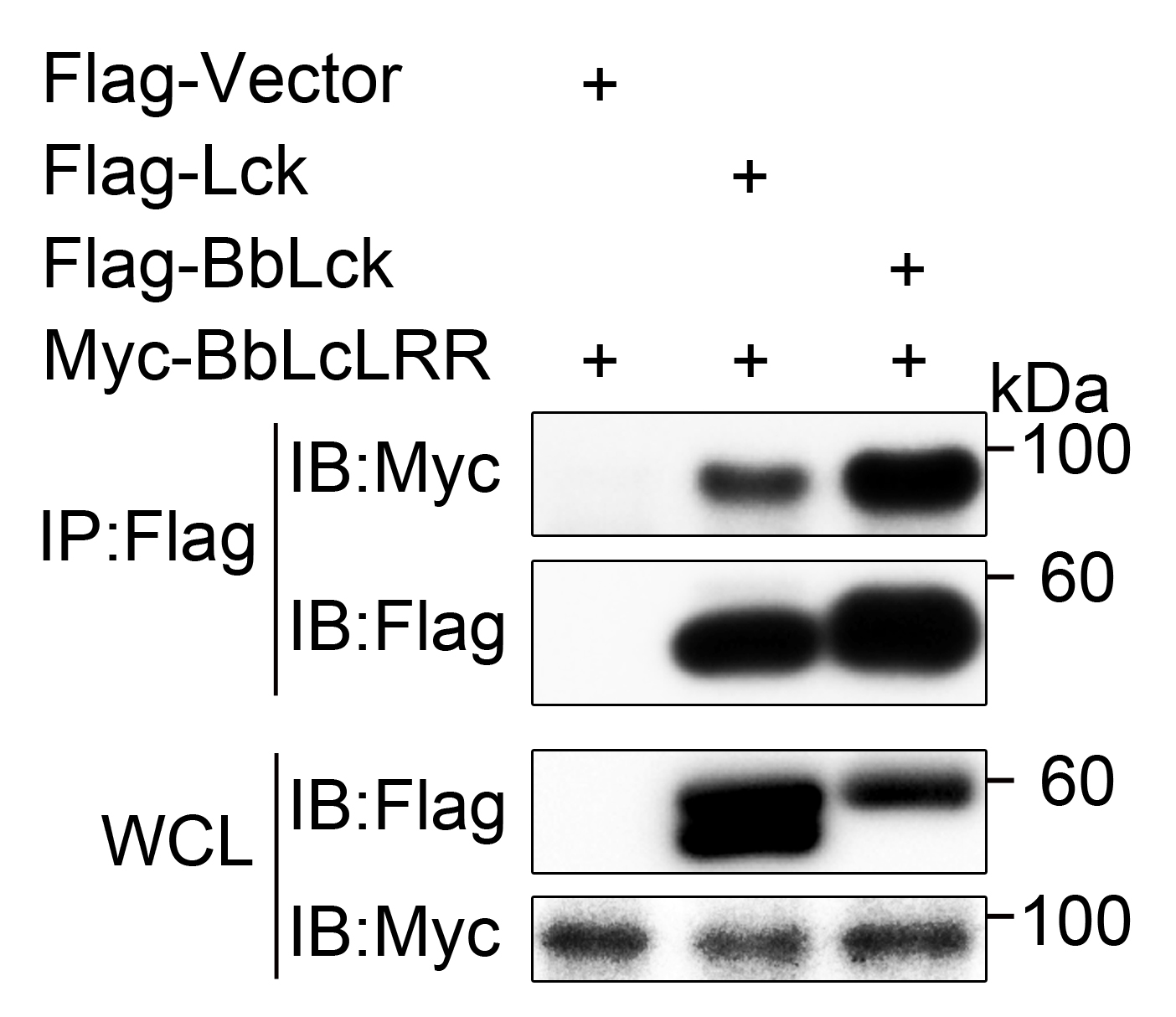

Supplement: Supplementary Figure 3 — Association of BbLcLRR with human or amphioxus Lck. The indicated plasmids were cotransfected into HEK293T cells, and cell lysates were immunoprecipitated with anti-Flag antibody followed by immunoblotting with the indicated antibodies. [file Image_3.jpg]

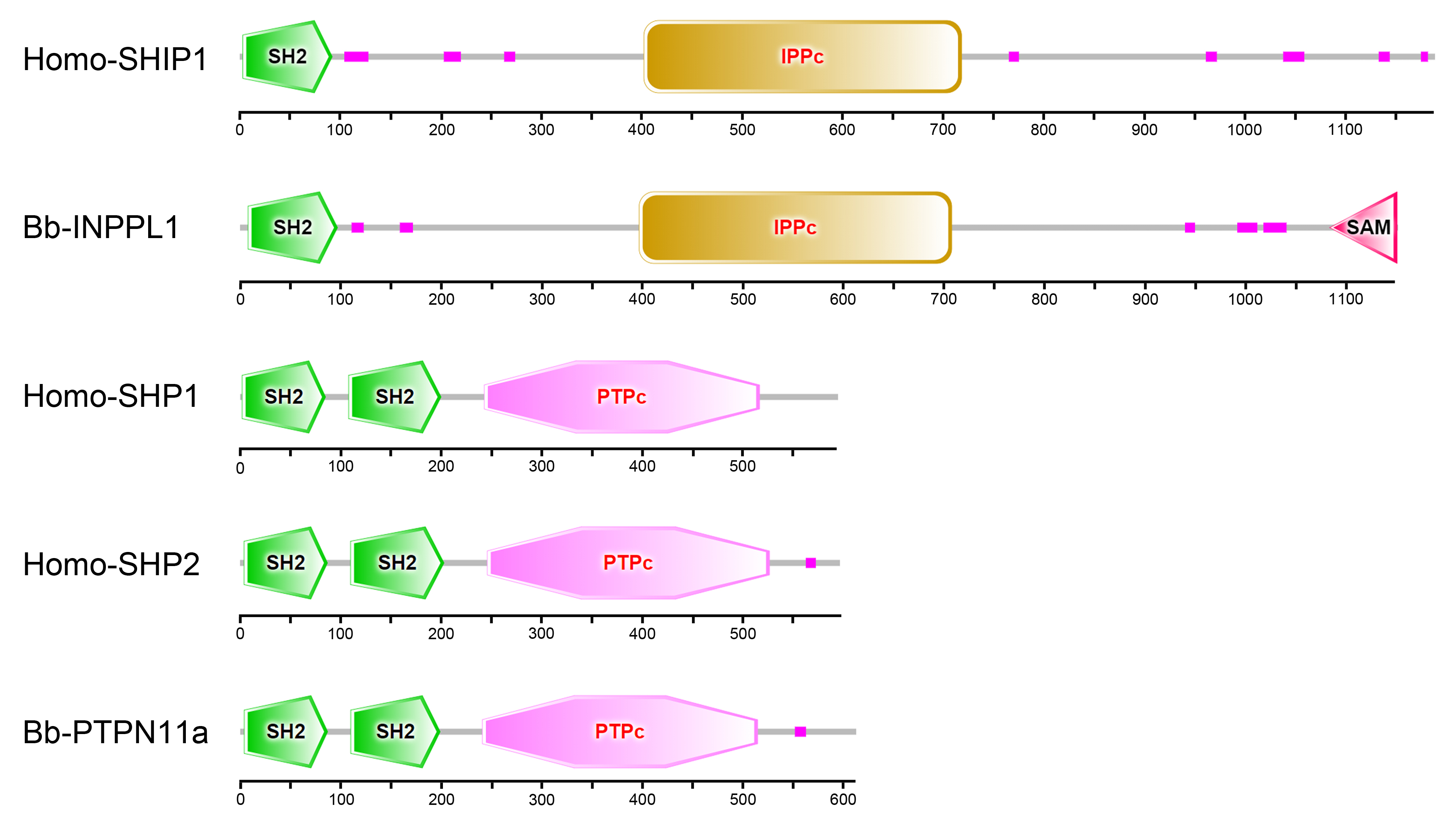

Supplement: Supplementary Figure 4 — Domain analysis of human SHIP1, SHP1, SHP2, and amphioxus Bb-INPPL1 (Accession No. XP_019617039.1), Bb-PTPN11a (Accession No. XP_019637720.1). [file Image_4.jpg]
